# Nature-based interventions in psychiatric inpatient settings

**DOI:** 10.3389/fpsyt.2026.1727355

**Published:** 2026-05-04

**Authors:** Julia Amann, Felix Bargfeld, Noah Augustin, Lydia Romund, Christian A. Müller

**Affiliations:** Department of Psychiatry and Neurosciences, Campus Charité Mitte, Charité – Universitätsmedizin Berlin, Berlin, Germany

**Keywords:** acute psychiatry, forest therapy, horticultural therapy, nature-based interventions, outdoor physical activity, psychiatric inpatients

## 1 Introduction

Beneficial effects of exposure to nature on various mental and physical health outcomes are well documented (1). Nature-based interventions (NBIs) utilize these effects for preventative, curative and rehabilitative purposes and encompass experiences and activities such as walking, gardening, and psychotherapeutic conversations in nature (e.g., known as forest bathing, gardening therapy, or walk and talk therapy).

A growing body of evidence indicates that NBIs are cost-effective and improve both physical and mental health (2, 3). According to a recent meta-analysis (3), NBIs lead to reductions in blood pressure as well as depression and anxiety levels. However, regarding psychiatric inpatient care–particularly in acute psychiatry–research on the effects of NBIs is sparse, while there are indications of potential benefits.

The aims of our article were to synthesize the current evidence on the effectiveness and safety of NBIs in psychiatric inpatient settings and to integrate the available evidence on their potential mechanisms of change in these contexts. In doing so, we sought to identify current limitations and avenues for future research.

## 2 Current state of research on NBIs in psychiatric inpatient settings

The two most recent systematic reviews and meta-analyses (3, 4) of the effects of NBIs on mental health included only one study (5), reportedly conducted with psychiatric inpatients. To the best of our knowledge, only four additional studies (6–9) were carried out explicitly in these settings, one of them in an acute care unit. Four of the studies had no control group and included predominantly depressed patients with sample sizes below 30. None of these studies specified which diagnostic classification system was used, nor was indicated that diagnoses were based on structured clinical interviews. Instead, diagnoses relied on clinical judgment, self-report measures, or the method of diagnosis was not reported. None of these studies collected long-term follow-up data. See **Table 1** for an overview of the methodology of these studies.

**Table 1:** Methodology of studies on nature-based interventions for psychiatric inpatients.

Study	Sample size	Diagnoses of participants	Diagnoses based on	Quantitative outcome measures	Qualitative data
Frühauf et al. (5)	14	mild to moderate depression, comorbidities excluded	clinical assessment, Beck Depression Inventory II (BDI II)	Feeling Scale (FS) Felt Arousal Scale (FAS) Mood Survey Scale (MSS)	none
Iwata et al. (6)	quantitative: 15 qualitative: 7, and a staff	depression, bipolar disorder and/or anxiety disorder and/or other diagnosis	not mentioned	Positive and Negative Affect Schedule (PANAS) Beck Depression Inventory (BDI) The Hamilton Depression Rating Scale – 21-item version (HDRS-21)	semi-structured interviews
Joschko et al. (7)	16	mild to severe depression, comorbidities e.g.: obsessive-compulsive disorder, post-traumatic stress disorder (PTSD)	Patient Health Questionnaire (PHQ)	Hamburg modules for assessing general aspects of psychosocial health for therapeutic practice, 49-item version (HEALTH-49): well-being subscale PHQ: depression, somatoform disorders and stress subscales Connectedness to Nature Scale (CNS)	semi-structured survey, field notes
Carlson et al. (8)	48	diverse: e.g., depression, anxiety disorders and PTSD; comorbid substance use disorder	not mentioned	none	semi-structured survey, field notes
Pieters et al. (9)	25	mostly depression, but also e.g., PTSD and gender dysphoria; comorbidities included	not mentioned	none	semi-structured interviews, field notes

### 2.1 Effectiveness of NBIs in psychiatric inpatient settings

Quantitative results document significant beneficial effects of NBIs on mood and well-being in psychiatric inpatients (5–7), measured using self-rating scales. With a sample of 15 patients with mood and/or anxiety disorders, Iwata et al. (6) conducted two-hour sessions weekly for 13 weeks in local forests in Ireland encompassing warm-up exercises, group walks and time to socialize afterwards. The authors documented a significant immediate reduction in negative affect and a significant increase in positive affect after the sessions as measured by the self-report scale Positive and Negative Affect Schedule. Changes in depression were assessed using both self-report and clinician ratings via Beck Depression Inventory and The Hamilton Depression Rating Scale and were analyzed descriptively only. Pre-post comparisons found average improvements of 36.8% and 54.3%, respectively. In a within-subjects experimental study, Frühauf et al. (5) also reported short term mood improvement in a sample of 14 depressed psychiatric inpatients. Each patient participated in three single 60-minute sessions: one session of a sedentary control condition, one session of an indoor physical activity, and a session of an outdoor physical activity. The latter resulted in significantly stronger improvements in activation and excitement measured using the Mood Survey Scale and the Felt Arousal Scale. A stronger improvement in affective valence measured using the Feeling Scale did not reach significance. Joschko et al. (7) implemented a four-week program consisting of three weekly 60-minute sessions in a nearby garden and forest including nature-based art, gardening, a gathering circle, and walks with a sample of 16 depressed inpatients with a diverse range of additional diagnoses (e.g., somatoform, eating, and anxiety disorders). They reported a significant decrease in depressive symptoms as measured via the depression subscale of the Patient Health Questionnaire and a significant increase in mental well-being as measured by the Hamburger Module zur Erfassung allgemeiner Aspekte psychosozialer Gesundheit für die therapeutische Praxis (see **Table 1** for the translated title). Additionally, a significant increase in nature connectedness was documented (measured via the Nature Connectedness Scale). Improvements on the somatoform disorders and stress subscales of the Patient Health Questionnaire did not reach significance. Overall, first quantitative results suggest mood improvements immediately after NBI sessions as well as after a complete program compared to baseline in psychiatric inpatients. However, the robustness of these preliminary findings is limited by the lack of control conditions, small sample sizes, and the use of heterogeneous patient samples whose diagnoses were not explicitly based on structured clinical interviews.

### 2.2 Mechanisms of change of NBIs in psychiatric inpatient settings

Based on semi-structured interviews and surveys as well as field notes, qualitative results regarding NBIs conducted with psychiatric inpatients lend support to potential mechanisms of change. One proposed mechanism involves cultivating a sense of meaning through purposeful action and social interaction (6–9). Joschko et al. (7) provided an example of a patient who enjoyed participating in the NBI because of the feeling to do something meaningful by helping both oneself and nature. Patients in the study conducted by Iwata et al. (6) pointed out that forest walks were meaningful to them as they created a sense of belonging to the therapy group. The feeling of making a meaningful contribution to a group, one’s own health as well as to nature by taking part in the NBI was also found by Pieters et al. (9) and Carlson et al. (8), who reported that these contributions also fostered a sense of agency and autonomy in patients.

Carlson et al. (8) provided 60-minute nature-based group therapy sessions in which patients focused on their senses while caring for and arranging plants indoors or in a nearby garden, complemented by conversations about previous nature experiences. In total, 75 psychiatric inpatients with diverse diagnoses (depression and anxiety disorders, post-traumatic stress disorder, and comorbid substance use disorders) participated in the program, 48 of them completed the semi-structured survey. Carlson et al. (8) concluded that the act of nurturing and observing growth in something they cared for fostered a sense of personal agency.

In the same vein, a feeling of self-efficacy was evident in statements of 25 patients in an acute psychiatric ward who participated in 45-minute NBI sessions including conversations about enjoyment of nature and subsequent nature-based art and gardening activities on the outdoor deck of the unit (9). Participants in the study were mostly diagnosed with major depressive disorder, an anxiety disorder or post-traumatic stress disorder, but some patients also suffered from bipolar or psychotic disorder.

Eliciting positive emotion, e.g. through revisiting pleasant memories and promoting physical activity, was identified as another working mechanism of NBIs in psychiatric inpatient settings (6, 8, 9). Participants in the NBI by Carlson et al. (8) reported that they regained access to positive memories of gardening. Similar results were reported by Iwata et al. (6) and Pieters et al. (9). Furthermore, Carlson et al. (8) highlighted that participants enjoyed the physical movement entailed in the nature-based activities.

Fostering calmness and relaxation emerged as another common theme of psychiatric inpatient reports on the effects of NBIs. Participants of nature walks provided by Iwata et al. (6) highlighted the calming effect of the quietness of the forest. Patients in an acute ward experienced spending time on the outdoor deck of the unit as calming (9). Caring for plants was reported to deepen that effect (9). Similar experiences were shared by participants in the studies of Carlson et al. (8) and Joschko et al. (7).

In summary, qualitative studies on NBIs in psychiatric inpatient settings provide preliminary indications that mechanisms of change include fostering meaning through purposeful action, promoting social interaction, and enhancing a sense of agency, notwithstanding the aforementioned limitations. Eliciting positive emotions via revisiting pleasant memories, physical activity and a sense of calmness emerged as another key mechanism across studies.

### 2.3 Safety of NBIs in psychiatric inpatient settings

None of the mentioned studies systematically collected data regarding adverse events of NBIs in psychiatric inpatient settings. However, participants in the qualitative studies reported that they felt safe and mostly comfortable during the NBIs (6, 7, 9).

## 3 Discussion

In conclusion, there is promising preliminary evidence for feasibility, efficacy, and safety of NBIs in psychiatric inpatient and acute care settings. First qualitative studies indicate that mechanisms of change of NBIs in psychiatric inpatient settings encompass promoting a sense of meaning, belonging and agency as well as fostering positive emotions. In this sense, NBIs could address concerns, which are often relegated in the face of acute clinical pressures and are aligned with person-centered and recovery-oriented approaches (10). Moreover, by fostering calmness, social connection and a sense of agency NBIs hold the potential to prevent escalation and thereby the use of coercive measures, since patient aggression in acute psychiatric settings can be triggered by agitation, frustration of social needs as well as a sense of powerlessness (11–13).

However, these conclusions should be considered tentative due to several limitations of the current evidence on NBIs in the psychiatric inpatient setting. These limitations include predominantly uncontrolled study designs lacking long-term follow-up and small samples consisting foremost of depressed patients who were not explicitly diagnosed via structured clinical interviews. Methodological shortcomings of this kind carry the risk of over- or underestimating the effectiveness of the interventions and hinder generalizability. The fact that most samples were not composed exclusively of depressed patients without comorbidities mirrors routine care conditions, thereby supporting ecological validity. However, this may also lead to an underestimation of safety risks, as it hinders the detection of potential adverse effects specific to certain diagnoses. Additionally, as structured documentation of safety risks has been missing, potential adverse events in general might have been overlooked to date.

Against this background, it is not yet possible to robustly evaluate the balance of benefits and risks. Accordingly, randomized controlled trials with sufficient sample sizes, long-term follow-up, and systematic safety monitoring across adequately diagnosed patient groups are needed to examine whether the implementation of NBIs in psychiatric inpatient settings should be further advanced.
